# Study of bypassing Microsoft Windows Security using the MITRE CALDERA Framework

**DOI:** 10.12688/f1000research.109148.2

**Published:** 2022-05-25

**Authors:** Nachaat Mohamed

**Affiliations:** 1 Assistant Professor of Homeland Security, Rabdan Academy, Abu Dhabi, United Arab Emirates

**Keywords:** APT, CPU, Attack, Exploit, Detection, Cyberattack.

## Abstract

**Background:** Microsoft Windows Security is a recently implemented safeguard for the Windows operating systems, including the latest versions of Windows10 and 11. However, there is a major shortcoming in this system to stop Advanced Persistent Threat (APT). These are government-financed groups that are funded to attack other government entities. Following the initial security breach, the hacked Windows device is used to access the rest of the network devices in order to transfer data to external storage (Exfiltration).

**Methods: **In this work, we have tested the Microsoft Windows Security system using MITRE CALDERA and ATT&CK frameworks and explain how APT groups are able to bypass Windows Security.

**Results: **In this study we used "54ndc47" agent through GoLang feature in MITRE CALDERA platform to test and bypass Microsoft Windows Security systems (MS Windows 10). Through it, we were able to bypass the Windows Security system and display entire files in the victim's device.

**Conclusions: **In this paper, we have provided recommendations to Microsoft to improve their Windows Security tool through the use of Artificial intelligence (AI).

## Introduction

Windows 10 and 11 incorporate Windows Security, which provides users with the most recent antivirus assurance. Windows Security will begin operating and secure the system from the minute one begins Windows. ^1^ It ceaselessly looks for malware (pernicious programs), infections, and security dangers. ^1^ In addition to this real-time assurance, overhauls are downloaded automatically to assist in keeping the device secure from ongoing threats. Since this mode is streamlined for tighter security, the Infection & Danger assurance zone has fewer alternatives. ^2^ Built-in security within this mode that naturally anticipates infections and other dangers running on user devices, and users then receive security overhauls as they continue using their device.

As with each previously released version, Windows 10 was intended to be the most secure Windows operating system. As part of that release, Microsoft presented Windows Security as a beneficial addition. ^3^ This offered an improved approach to building, sending, and adjusting Windows data, and unused highlights are built persistently with each overhaul. Windows 10 also has more layers of assurance that assist in securing organizational information, as well as identifying unsafe behaviors and modern assaults. Windows 10 is therefore making a difference using superior secure data and with each subsequent release, Microsoft have built upon the existing security measures by including modern security highlights. ^3^ They have consciously tended to dangers through iterative design, ensuring that improved security is one of the operating system’s greatest benefits. ^4^^,^^5^ However, there is a huge shortcoming in this product, and traditional hackers and Advanced Persistent Threat (APT) groups are utilizing varying methods to bypass this improved level of Windows security.

In order to test the level of vulnerability, we simulated an APT attack to bypass the Windows security, using the CALDERA framework from MITRE. CALDERA is a tested framework to evaluate features of infrastructure security posture through penetration testing. It tests the entire suite of tactics and techniques used by APT. ^12^^,^^13^ The CALDERA framework is used by red teams to protect organizations against sophisticated attacks, and adversary emulation is resource intensive and can present challenges. To combat these challenges, the CALDERA framework offers an intelligent, automated system of red teamwork, which can reduce the resources needed by penetration testing and security teams for routine testing. This then leads to providing the right solution/recommendation to the blue teams/organizations. ^14^^,^^15^

CALDERA can also be utilized to test endpoint security arrangements and evaluate a network’s security capability to withstand the common post-compromise, antagonistic strategies contained within the ATT&CK approach. ^14^ CALDERA leverages the ATT&CK approach to distinguish and imitate enemy behaviors as if a genuine interruption is happening. This empowers computerized evaluations of a network’s defenselessness to enemy penetration, permitting organizations to see their systems through the eyes of a progressive, determined danger, on-demand and to confirm the strength of guards and security arrangements currently based upon known risk methods. It also employs an enemy representation dialect; the ATT&CK profile. This is a motor choice to prepare assembled information and select ensuing activities, and a specialist conducting the operation. Utilizing CALDERA can therefore decrease assets required for appraisals and permit groups to focus on modern approaches to more difficult issues. ^16^ This can enable organizations to tune behavioral-based, interruption discovery frameworks more quickly as they are deployed. ^17^

CALDERA is also complementary to other types of security evaluation. The infrastructure security position is commonly surveyed based on program fix levels, security controls, and shield devices. Whereas numerous interruption location apparatuses depend on looking for known risk markers which alter as often as possible. Appraisals and enemy discovery are only typically based upon foe behavior. ^18^ This can change how shields respond to, identify, and react to dynamic dangers. CALDERA can also make a difference to shield approaches as it can move past discovery of pointers of compromise, through to location and reaction of foe behavior. ^17^ In addition to the expansion to the open-source adaptation of CALDERA, Miter maintains a closed-source form that highlights extra capabilities, creating superior adaptability to more endpoints. These are used to examine authorizing or collaboration exercises on closed-source CALDERA. ^18^^,^^19^

### Related work

Microsoft’s Protector is proficient at recognizing malware records, blocking misuses and network-based assaults, and hailing phishing destinations. It incorporates basic PC execution and wellbeing reports, as well as parental controls with substance sifting, utilization impediments, and area following. Antivirus software is fundamental if the user is utilizing a Mac or Windows device; both come with some level of infection assurance built in. ^3^^,^^4^^,^^7^ In order to build upon these protections and develop endpoint security, protection against malware and possibly undesirable programs, it is best to introduce a third-party antivirus application. Microsoft Guard was not present in the old versions of Windows operating systems, so users of Microsoft operating systems purchased or acquired an antivirus program for device protection. This has changed over time, and Microsoft has integrated an antivirus program to protect its operating systems and software running under them 10. ^8^^,^^9^

Microsoft now indirectly implies that when in the Microsoft system work environment, a user does not need the support of other companies for protection. Microsoft are now able to provide this protection to the end user utilizing Microsoft Protector. A user’s device, files, and data are under its protection from direct or indirect tampering. ^10^^,^^11^ In addition, it is now true that Microsoft Shield is sometimes seen as a competitor with decent protection capabilities when compared to the large security systems in the free antivirus world. ^10^ This product has evolved significantly since being first developed due to Microsoft’s relentless pursuit of this product. This could be because it would prevent the company’s end users from buying protection products from other companies, which was clearly noted in the latest evaluations conducted by the main independent laboratories that conduct tests at fixed intervals to measure the readiness of antivirus applications. ^7^ A test of these available antivirus systems was conducted in July and October 2020 by AV-Comparatives, showing that Microsoft’s performance improved considerably, and Microsoft was rated overall as ‘good’ as it was able to stop 99.5% of the risks, and in this test it came in twelfth place among 17 competing anti-virus programs. ^22^^,^^23^ In another similar evaluation conducted by SE Labs, Microsoft’s product scored 99%, making it the fifth out of 13 participants in the competition. In a further report on protection against intrusion, as of 2020, Microsoft ranked fourth; a result indicating its quality, especially considering the competition was with top providers in the antivirus industry. ^24^ The overall picture, therefore, is that Microsoft Guard is now more than robust in terms of protecting the user from hacking and malware. In short, according to Avira, we can say that Microsoft Protector may be sufficient for the user to satisfactorily protect their data, but it is also clear that it represents a reputable and reliable ‘non-free’ antivirus option. ^25^

With regards to users who do not have high levels of technical experience, it is difficult for them to correctly judge whether this product is sufficient to protect them or not. It can therefore represent an advantageous product for some, and a limited one for others. This difference depends on the way users navigate their computer hardware, software and the Internet. ^8^ However, if the user can easily get a free protection program that meets their needs and does not put them at risk, then those can represent a suitable subscription for both experienced users and novice users. ^7^^,^^8^^,^^10^ Unfortunately, the area of antivirus software can also be used by malicious groups to distribute agents used to penetrate users’ systems. This has and will continue to occur, and therefore improved access to reputable antivirus programs for both ends of the market is a positive change. ^11^ However, it is also true that although Microsoft strives to maintain its reputation and increase the number of its users by providing products that meet user needs, ^30^ the findings from this paper prove with conclusive evidence that it is possible to bypass Microsoft Anti-Virus and take control of the device, just as the same device can then be used to hack the rest of the devices in the same infrastructure.

## Methods

The objective of robotized/simulated APT attacks imitating adversaries (APT groups) is to detect weaknesses in the current systems and provide system defenders with an apparatus able to execute a full-scale evaluation of their organization, working in a way that is comparable to a genuine adversary. Such an apparatus has noteworthy utility for guards, ultimately providing a standard for what their network looks like to an enemy, producing preparedness data, identifying shortcomings and/or misconfigurations, and testing in-place security measures and devices. This provides a valuable experimental proof for a cautious blue team to build on. ^2^^,^^31^ We differentiate this with a device that, for example, only distinguishes assault methods and approaches without actually executing them. This tool could provide an outline of what the organization looks like digitally, but typically will come up short to realize other utilization cases because it omits critical, hard-to-measure points of interest, and requires the authenticity of genuine execution. The objective of CALDERA therefore is to drive automated adversaries that do not just imitate, but also incorporate:

1) Selecting and chaining actions in ways comparable to how an attacker would.
2) Allowing shields to be able to utilize the tool without requiring express arrangement of points of interest. These are both time-intensive to gather and are nearly incomprehensible for guards to completely track.
3) The framework executes the same techniques that a genuine enemy would, and, like a genuine adversary, should begin at initial compromise, and progress to their intended end after achieving (or coming up short to realize) a particular set of goals.
4) Clients of the framework ought to be able to run appraisals with methods of their choosing, as well as have the capacity to include modern strategies. ^2^^,^^30^^,^^32^

CALDERA therefore offers and adds to the field of information security, as it automates the penetration-testing process and simulates the tactics and techniques used by state-funded hacking groups to attack other countries for the purposes of espionage or sabotage. ^16^ As a consequence, it is used as a tool by red teams to simulate a real attack, and blue teams can then use realistic data to explain protection plans and methods to protect public and private institutions. ^18^ It has also been designed to deal with aspects of the defensive and offensive systems of MITER ATT&CK. This system consists of two components: 1) the server that contains all the operations with an application interface to control all the operations that take place on the victim’s device. ^18^ 2) Additions. This is the main user interface through which it is possible to control the addition and deletion of components that serve the attack process, which must eventually lead to access and control of the largest possible amount of the target device’s resources. ^19^^,^^20^ The end result therefore provides a product that will automatically test an organization’s infrastructure against the tactics and techniques used by currently operating hackers. ^15^ It provides the red team a contemporary and informed ability to test the dangerous infrastructure in the shortest possible time and with as few people as possible, so one or two individuals can simulate an entire team of penetration testers. This is provided by CALDERA, produced and developed by MITER. ^17^^,^^32^^,^^37^ It can be directly used to design a client and test it on potential victims to test if there is a vulnerability that hackers can use against the targeted network and the rest of the network devices. ^16^^,^^33^ This paper presents an application of the CALDERA approach to Microsoft’s security systems. It demonstrates that the protections were bypassed, new permissions were added, and all files on the victim’s device were accessed.

The client used in the attack is called Sandcat, a small command generation program identified as “54ndc47”, which can evade and attack the opponent, and reconnect with the CALDERA server. This “54ndc47” agent was created in GoLang to be compatible with most existing operating systems. The operation of “54ndc47” requires port 8888 to be opened to communicate with the server. To run “54ndc47”, one of the commands included in this framework that corresponds to the operating system or the so-called potential target is used, which allows the user to run remote commands. ^15^ These commands download the “54ndc47” executable compiled and provided by CALDERA and immediately run it on the victim or target machine. All commands and instructions can then be accessed through the Sandcat plugin. Once commands are executed on the target device, the attacker’s device appears to have successfully communicated with the victim’s device after sending it to CALDERA. With regards to the CALDERA server, every time a transfer command is run, the attack command forcefully reassembles itself and changes its source code to contact the attacker’s machine, so that it gets a “MD5” miscellaneous registry hash. This certainly helps bypassing command-dependent signature detections in files. ^17^^,^^38^ When running “54ndc47”, important parameters can be used after the executable is running. The agent in this regard must be sent to at least one victim device within the target infrastructure. ^19^ In addition, virtual groups will be used during the attack process and the communication between the victim’s device and the attacker. The attack process is organized and arranged to maintain the lightweight code, “54NDC47” or hack commands which are sometimes restrictive to avoid detection devices in general.

The work used in the attack process has powerful additional features, referred to as GoCat extensions. It also includes an extension to the existing GoCat module tokens to provide many benefits, such as the use of a peer-to-peer broker, additional proxies, and additional C2 communication protocols. ^13^ To request other restricted plugins from GoCat, the client can perform a HTTP merge of all GoCat extensions when C2 is queried, which is called a custom assembler. The title should be a comma-separated list with technical considerations. ^34^ The server includes additional extensions and is not required if their conditions are met (e.g., if extension A requires a specific GoLang that is not available on the server, then extension A is almost certainly not included at this point). It is possible to set default values for these alternatives when Sandcat is pulled from an attacker’s machine. Of course, this is highly valuable if hiding parameters from a method is required. This can be done by passing the values as headers instead of as parameters.

For illustration, the following will download a windows executable that will utilize http://192.168.1.14:8888 as the server address rather than http://localhost:8888.

**Figure 1.**
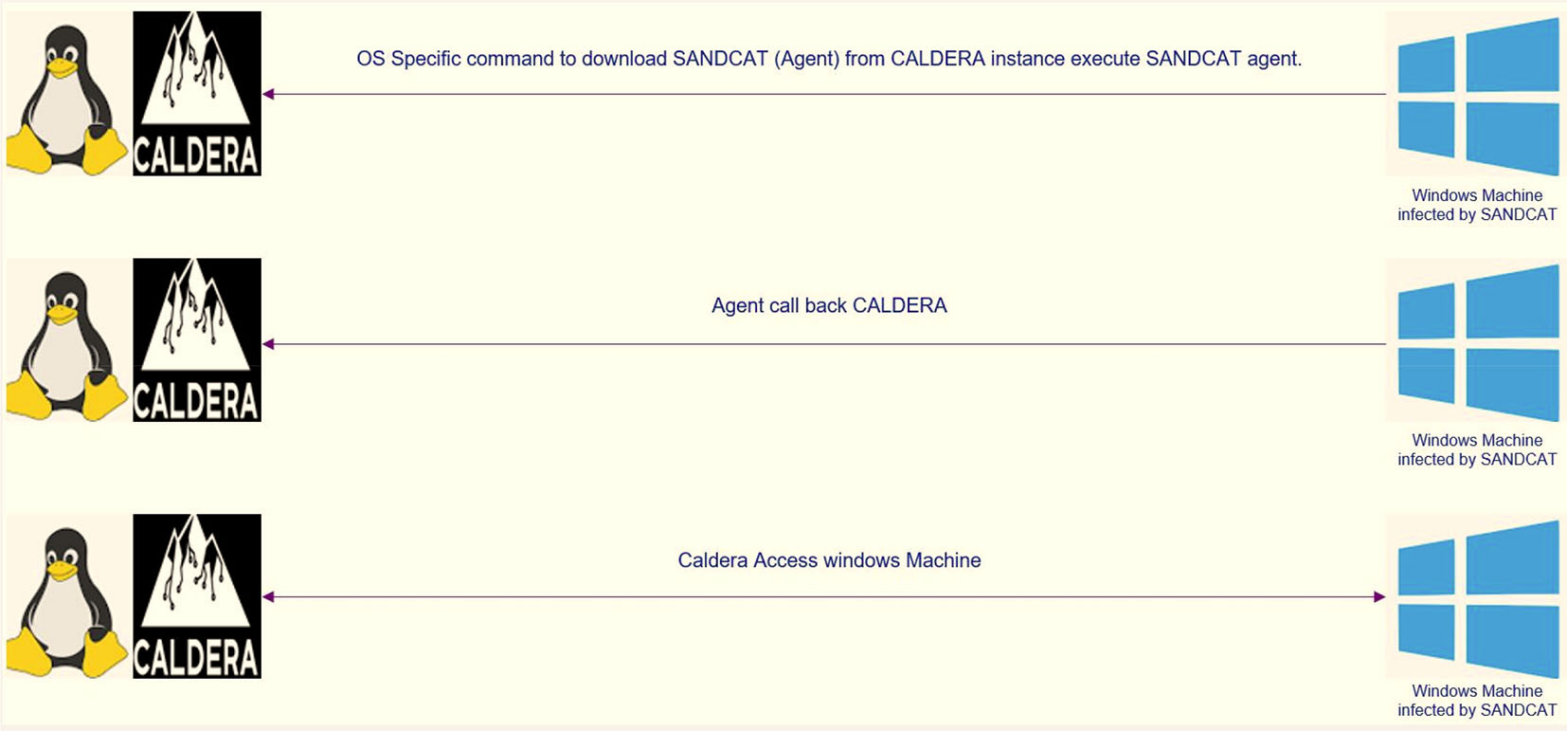
SANDCAT methodology (agent).

### Steps

*First step: update Kali Linux*

**Figure 2.**
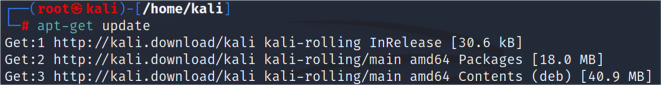
Updating Kali Linux.

In this step, we updated the Kali Linux system version 2022.1 to get the latest version of all the programs in the Kali Linux distribution; this can be done by typing the following command in the terminal “apt-get update”. Figure 2 shows the command used to update Kali Linux.

*Second step: installing CALDERA*

**Figure 3.**
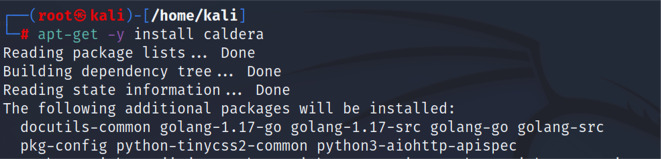
Installing CALDERA.

Then, we proceeded to installing the CALDERA framework within the Kali distribution by using the following command “apt-get -y install caldera”. Figure 3 shows the command used to install CALDERA.

*Third step: running CALDERA*

**Figure 4.**
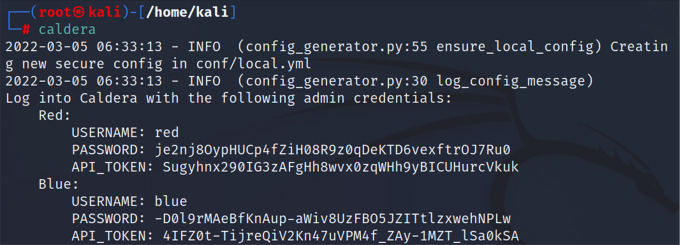
Running CALDERA.

Since CALDERA was integrated into the Kali repositories of the latest versions, this made it easy to install and run the CALDERA system from Kali Linux. After we installed CALDERA in the previous step, we then ran it via the command “caldera”. Figure 4 shows the command used to run CALDERA.

*Fourth step*

After using the previous command, we obtained the login information to the CALDERA framework, red username and password, blue username and password. In this study, we logged in using the red user and password. Considering that CALDERA runs through port 8888, we could start CALDERA by going to the browser and typing the IP of the CALDERA device followed by the port as in the following example. http://192.168.0.14:8888/ or http://localhost:8888/

Red:

 USERNAME: red

 PASSWORD: je2nj8OypHUCp4fZiH08R9z0qDeKTD6vexftrOJ7Ru0

 API_TOKEN: Sugyhnx290IG3zAFgHh8wvx0zqWHh9yBICUHurcVkuk

We then logged into the CALDERA framework, and selected "Navigate", then "Agent", and then we chose the orange key on the left side “Click here to deploy an agent”; after that, we identified the agent kind “54ndc47. Finally, we specified the operating system as “Windows”. Depending on these settings, the CALDERA system generates a code to create a client to bypass the Windows Security system and get a direct connection to the victim’s machine as shown below:

($server="192.168.0.14:8888/";$url="$server/file/download";$wc=New-Object System.Net.WebClient;$wc. Headers.add("platform","windows");$wc. Headers.add("file","sandcat.go");$data=$wc. DownloadData($url);$name=$wc. ResponseHeaders["Content-Disposition"].Substring($wc. ResponseHeaders["Content-Disposition"].IndexOf("filename=")+9).Replace("`"","");get-process|? {$_.modules.filename -like "C:\Users\Public\$name.exe"}|stop-process -f;rm -force "C:\Users\Public\$name.exe" -ea ignore;[io.file]::WriteAllBytes("C:\Users\Public\$name.exe",$data)|Out-Null;Start-Process -FilePath C:\Users\Public\$name.exe -ArgumentList "-server $server -group red" -WindowStyle hidden;)).

### Ethics approval

This study was approved by Rabdan Academy (Homeland Security (HLS) department), Abu Dhabi, United Arab Emirates.

## Results

### Bypassing Windows security

Organizations may fail if they do not implement a solid approach against apt attack and confirmation controls may then permit an aggressor to evade verification. In addition, enemies may also evade the verification component through taking substantial victim sessions and cookies. It is also possible to avoid authentication powerlessness, which appears to permit assailants to perform different noxious processes by avoiding the device verification method. After performing this process, the primary concern is verification bypassing abuse, solely because of a powerless confirmation structure. Companies that fail to maintain a robust and secure infrastructure may create many conditions that allow an attacker to bypass verification. ^5^^,^^6^^,^^38^

**Figure 5.**
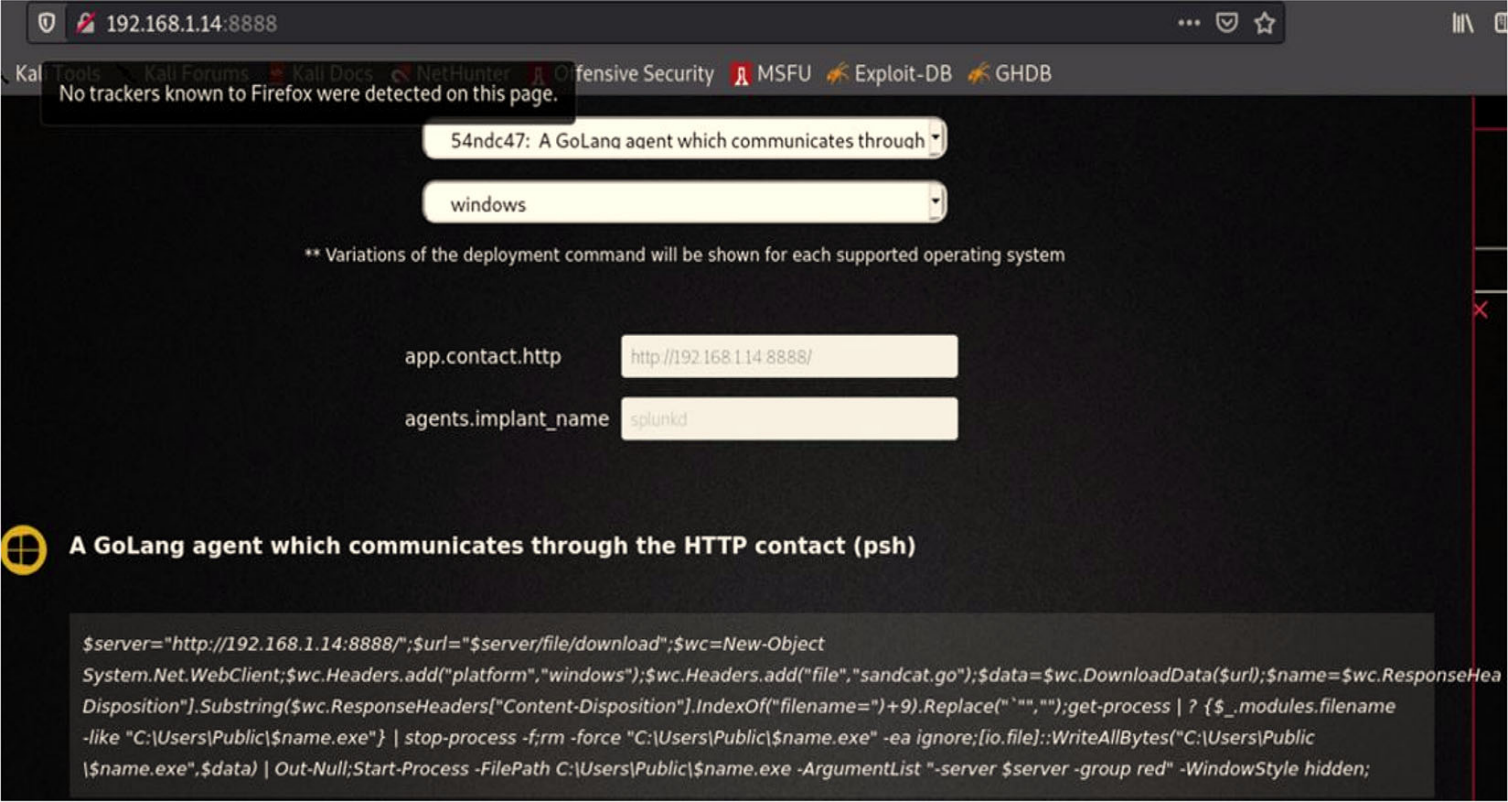
Creating an agent for a specific OS.

In this study, CALDERA was used to send a single command to the victim’s device, and through it enable the opening of a session on the target device. This provided the hostname, username, privilege, group, and other sensitive information. Figures 3 and 4 show the command used to bypass Microsoft Windows security and take over the victim machine through got active session over victim machine. Figure 5 shows create agent from CALDEARA server for Windows operating system.

**Figure 6.**
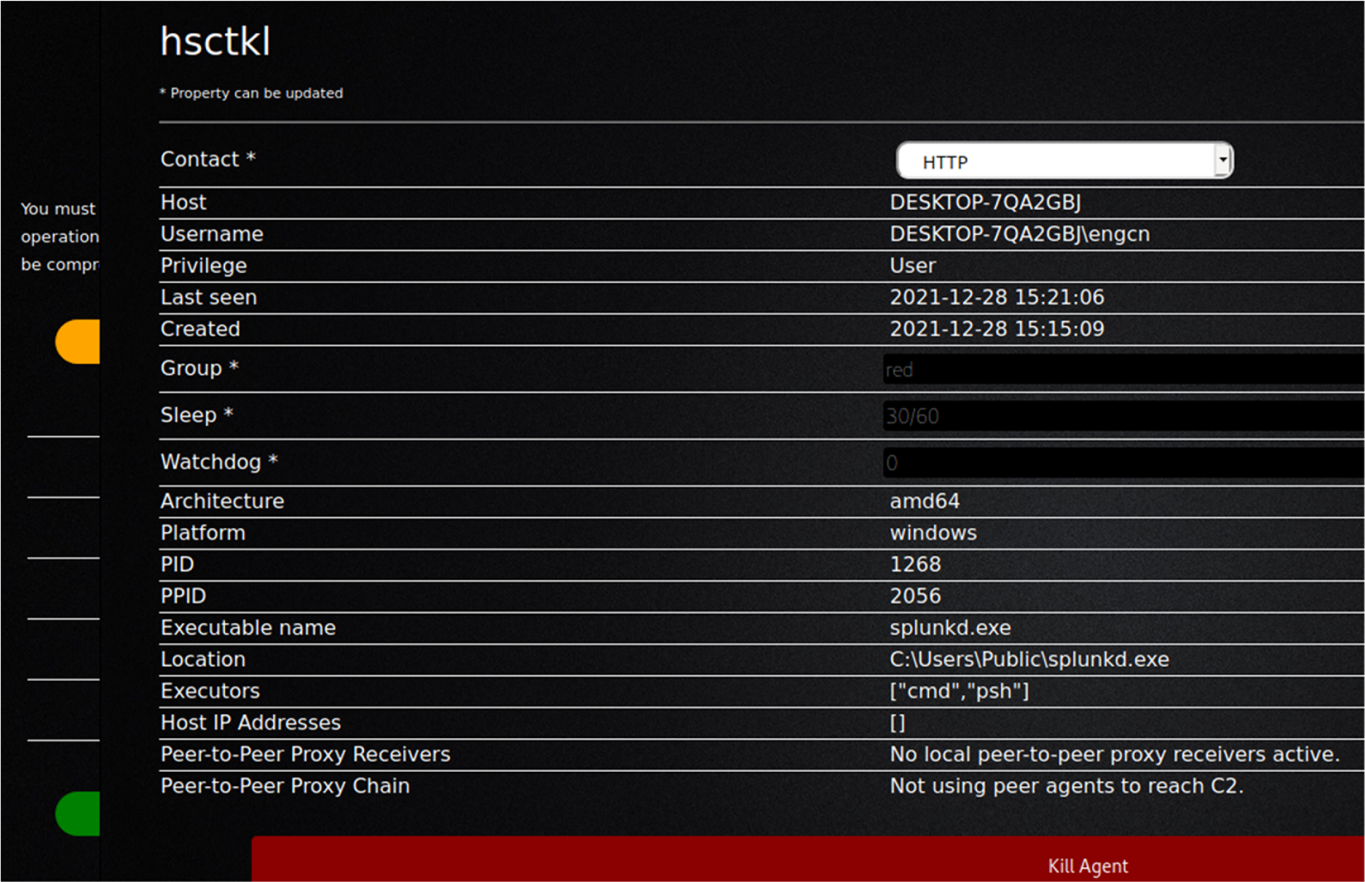
Bypassing Windows Security (connected with victim machine and take over through the agent).

Following this, the green color batten was utilized (agent 16232) to open the operations screen; this provides considerable possibilities with regards to implementing all the tactics and techniques used by the offensive groups’ APTs. All this was performed without any warnings or messages identifying suspicious activity on the victim’s device, even though the version installed was recently updated to the latest one available. Figure 6 shows bypassing Windows security (connected with victim machine and take over through the agent).

**Figure 7.**
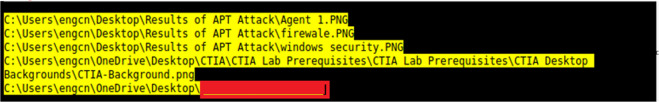
Results from the victim machine (paths to aggregated files).

After bypassing the Windows security system by agent that was created by CALDERA and then applying the Collection tactic, gives us the ability to collect the entre files from the victim’s machine as shown in the following figure. Taking into account that attacker can use the same device to penetrate the rest of the devices and servers in the target infrastructure trough applying lateral movement tactic, credential access tactic, credential dumping technique. Figure 7 shows results from victim machine (paths to aggregated files).

The next figure present example of results (paths to aggregated files).

As we can see, these paths have become compromised, and all files can be transferred to the attacker’s device.

C:\Users\engcn\Desktop\Results of APT Attack\

C:\Users\engcn\OneDrive\Desktop\CTIA\CTIA Lab Prerequisites\CTIA Lab Prerequisites\CTIA Desktop

C:\Users\engcn\OneDrive\Desktop\

The following figures shows the final report that was generated by CALDERA after bypassing Windows Security and getting files from the victim’s machine as shown earlier. The full report has been uploaded to the GitHub website as shown in the Data Availability section. The entire code for this study has been uploaded to GitHub and archived in Zenodo.

**Figure 8.**
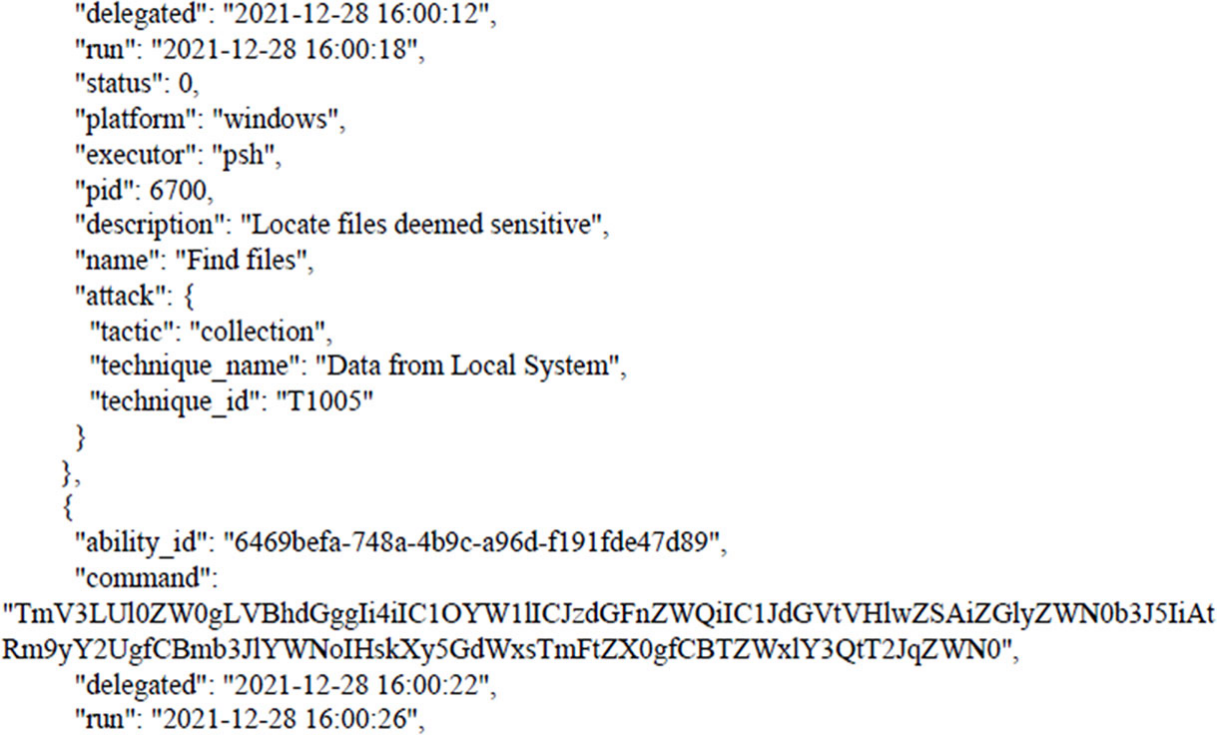
CALDERA commands/report.

**Figure 9.**
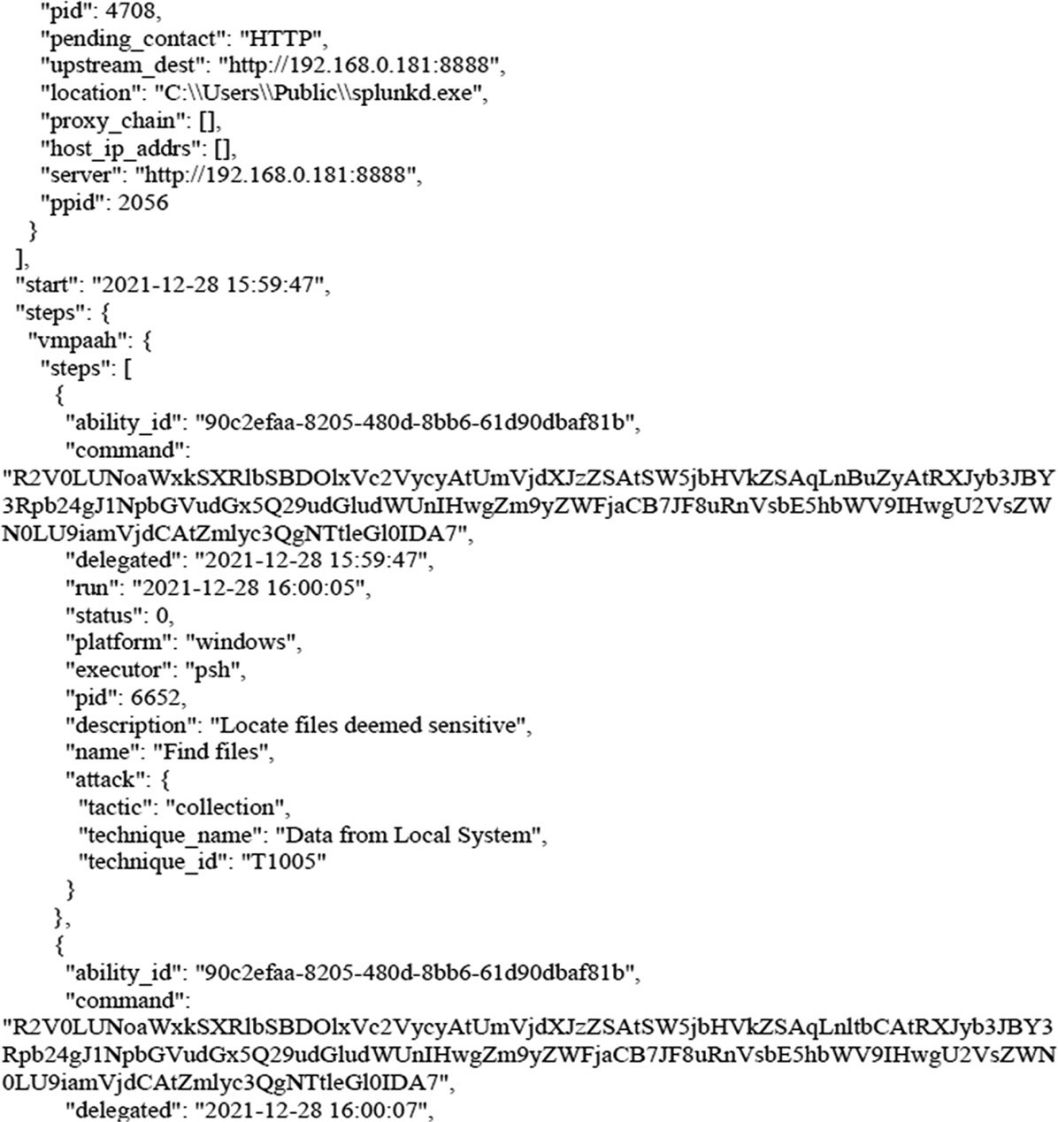
CALDERA commands/report.

## Discussion

### ATT&CK

**Figure 10.**
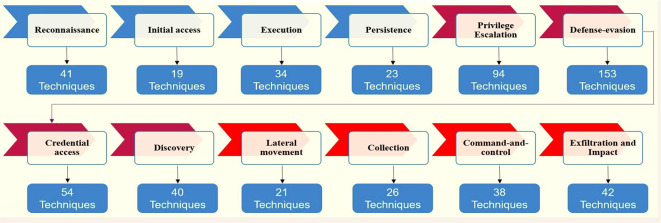
ATT&CK tactics and techniques.

Adversarial Tactics, Techniques, and Common Knowledge (ATT&CK) is a database used by information security professionals to understand the methods and techniques used by high-level attack groups utilizing APT, and these groups may be funded by state actors to attack other countries or regions. ^37^ ATT&CK allows to develop new methods and plans to protect against attack groups. The most important thing distinguishing ATT&CK is that it is free and available for governments and private organizations. ^35^ It is possible to take advantage of this approach in order to develop interception methods and defensive plans against offensives by attack teams funded by state actors for espionage or sabotage purposes. ^36^ ATT&CK contains 14 tactics and more than 500 techniques to counter the attacks of these groups. ^38^ Figure 1 shows which tactics and techniques are designed based on MITER. Figure 10 shows ATT&CK tactics and techniques.

### Real scenario

**Figure 11.**
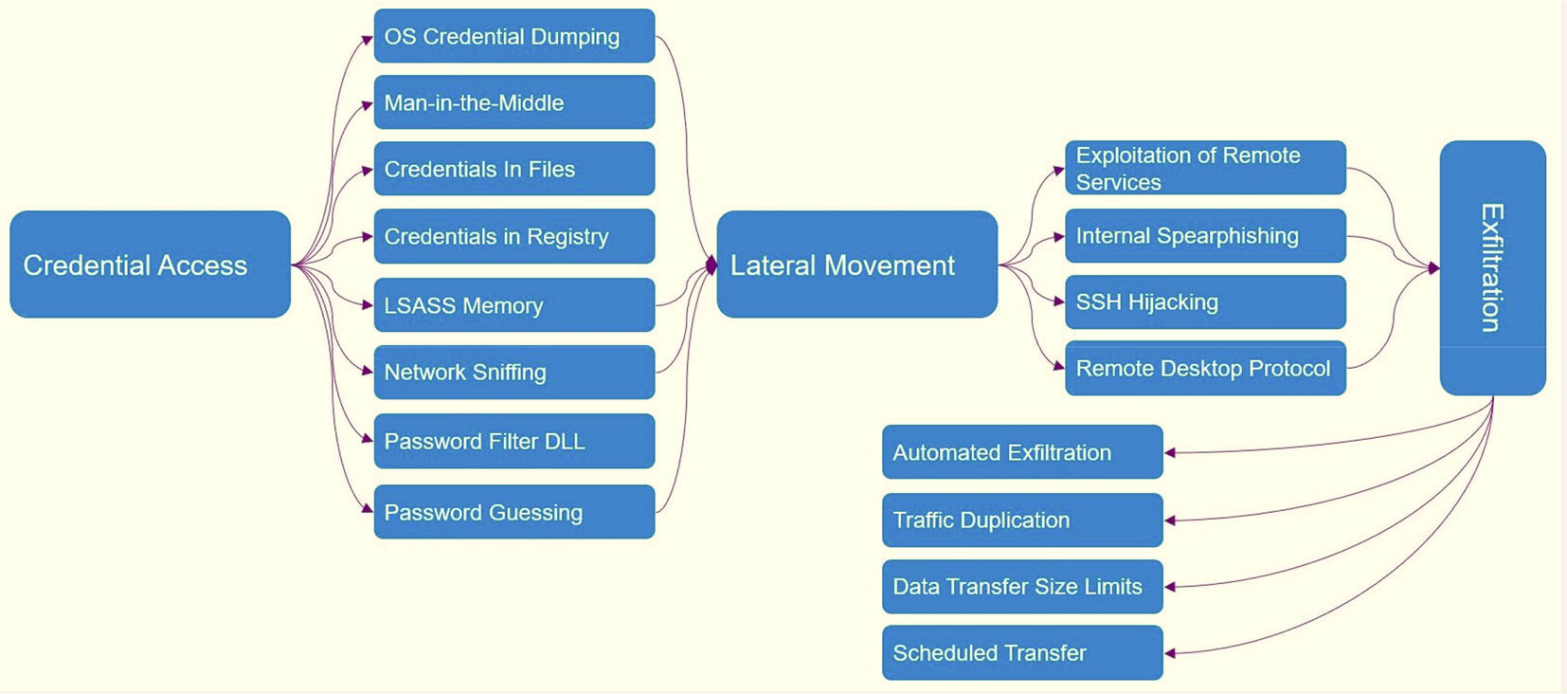
Windows defender, and firewall cannot detect the payload.

In this scenario, Figures 8 and 9 illustrates how, through the application of CALDERA, it was possible to infiltrate all the data from the victim’s device after achieving the initial access, credential access, and using lateral movement tactics and techniques. Through using the indicial access (IA) tactic, the enemy attempts to create a ‘foothold’ inside the existing infrastructure to allow access into the target network. IA tactics are ultimately used to comprise the target infrastructure that access different passage paths, in order to establish an introductory, solid footing inside an arrangement. The strategies utilized to pick up a dependable balance incorporate a focus upon spear-phishing and abusing shortcomings on web servers’ interfaces. The privileges that were obtained at the beginning of the penetration stage greatly facilitated the upgrade process to obtain full privileges on the victim’s device, then completely manage the victim’s machine and use it to access the rest of the network resources. This may result in limited or no use after passwords have been changed. In this scenario, spear-phishing was conducted through normal commands, sent to the victim machine from the CALDERA attacker machine, in order to pick up get to casualty frameworks. Spear-phishing by incomes of value may be a particular variation of command execution. It is as diverse as most of the more common methods of spear phishing, in that it engages in taking advantage of third-party administrations, rather than specifically using project mail channels. All spear-phishing forms are electronically conveyed social building focused on a particular person, company, or industry. In this situation, enemies send messages through different social media administrations, individual webmail, and other non-enterprise-controlled administrations. These administrations are more likely to have a less-strict security arrangement than other undertakings. As with most types of spear-phishing, the objective is to create affinity with the target or develop the target’s intrigue and attention in a variety of ways. Figure 11 Windows defender, and firewall cannot detect the payload when APT used ATT&CK against target infrastructure.

## Conclusions

This paper focused upon the application of the MITRE CALDERA framework to test Microsoft Windows security. Utilizing this framework, the paper explains and provides evidence on how to bypass Microsoft Windows security. A primary recommendation following this research, is that Microsoft specifically work to improve the level of security in recognizing commands written on devices by using artificial intelligence. The primary method for this can be through analyzing the commands before executing them. Finally, the paper advises researchers to study Tactics Techniques and Procedures (TTPs), MITRE CALDERA, Artificial Intelligence, Machine Learning, and Deep Learning as a future work to develop security solutions against APT attacks.

## Data availability

### Underlying data

All data underlying the results are included as part of the article and no additional data are required.

### Extended data

Zenodo: Nachaat3040/CALDERA-Code-Bypassing-Microsoft-Windows-security-: Cyber Attack DOI: https://zenodo.org/record/6309927

This project contains the following extended data:

- Agent 1.png
- CALDERA Code - Bypassing Microsoft Windows security.txt
- Collect info blugin 2.png
- Files collected 4.png
- README.txt
- collect txt files 3.png

Analysis code available from: https://github.com/Nachaat3040/CALDERA-Code-Bypassing-Microsoft-Windows-security-/releases/tag/CALDERA

Archived analysis code as at time of publication: https://zenodo.org/record/6309927

License: MIT
