# Supplementary material for: Study of bypassing Microsoft Windows Security using the MITRE CALDERA Framework
Source: F1000Res. 2022 Sep 29;11:422. Originally published 2022 Apr 14. [Version 3] doi: 10.12688/f1000research.109148.3 (PMC9525993; doi:10.12688/f1000research.109148.3)
Supplement: Peer review history

# Peer review: Reviewer response for version 3

**DOI:** 10.5256/f1000research.138325.r151867

**Published:** 2022-09-30

**Authors:** Hamed Taherdoost

**Affiliations:** 1 Research Club, Research and Development Department, Hamta Group, Hamta Business Corporation, Vancouver, BC, Canada; 2 University Canada West, Vancouver, BC, Canada

Thanks

Is the work clearly and accurately presented and does it cite the current literature?

Yes

If applicable, is the statistical analysis and its interpretation appropriate?

Not applicable

Are all the source data underlying the results available to ensure full reproducibility?

No source data required

Is the study design appropriate and is the work technically sound?

Yes

Are the conclusions drawn adequately supported by the results?

Partly

Are sufficient details of methods and analysis provided to allow replication by others?

Yes

Reviewer Expertise:

Cybersecurity, Information Security Management, Blockchain, Technology Adoption and E-Service

I confirm that I have read this submission and believe that I have an appropriate level of expertise to confirm that it is of an acceptable scientific standard.

# Peer review: Reviewer response for version 2

**DOI:** 10.5256/f1000research.134330.r146866

**Published:** 2022-08-18

**Authors:** Hamed Taherdoost

**Affiliations:** 1 Research Club, Research and Development Department, Hamta Group, Hamta Business Corporation, Vancouver, BC, Canada; 2 University Canada West, Vancouver, BC, Canada

This is a commendable work and a lot of effort has been put into it. However, it includes some defects that are required to be modified before being published. The comments are listed as the following:

- In Keywords, it is better to include more keywords especially those frequently appeared in searches in this field.
- The paper contains some grammatical, punctuation and typographical errors. It requires minor revision. It would benefit from a thorough language edit to make it easier to follow. Some instances of errors are as the following:
   
  - Missing punctuation in the third paragraph: **In this study (In this study,)** we used "54ndc47" agent through GoLang;
  - Grammatical error in singular and plural form: Microsoft **have (has)** built upon the existing security measures by including modern security highlights.
  - Spelling error: Appraisals and enemy discovery are only typically based upon **foe (for) **behavior.
- As this study is conducted based on sending a single command to the victim’s device, different results may be concluded by changing commands. Besides, this is dynamic field as attacks and defense layers are changing constantly. It is recommended to add the limitations of the study to clarify the conditions of the study and the make the study free from any bias or directions.
- It is concluded that use of Artificial intelligence (AI) improves the Windows Security tool; however, enough background is not provided to link the concept and clarify the impact of AI in this case. The existing gap in this area that can be addressed using Artificial intelligence should be clearly defined to justify the audience regarding the correlation between concepts of the study and the necessity of applying AI. Thus, it is recommended that the author adds a brief statement introduction in the beginning of the study about AI or other possible solutions based on the study.
- The motivations for using MITRE CALDERA and ATT&CK frameworks in this study are not clearly defined in the beginning of the study to attract the reader about the existing gap and the necessity of the study and clarify the question of the study. In terms of the substance of the abstract, it is recommended to consider emphasizing the motivation for the study to engage the reader in the very beginning of the paper.
- The paper also fails to identify new directions. The manuscript considers papers on aspects of the selected topics that are not new, that are not new directions in these topics, but rather rehashing of aspects already research and published in early years.
- The paper also fails to identify future directions for other studies in this area. The manuscript considers a method and its relevant steps appropriately; however, it is recommended to add future directions for employment of other methods in this area.
- In the related work section, the author should compare other works with the proposed one, concluding why this study is a must in this community.

Is the work clearly and accurately presented and does it cite the current literature?

Yes

If applicable, is the statistical analysis and its interpretation appropriate?

Not applicable

Are all the source data underlying the results available to ensure full reproducibility?

No source data required

Is the study design appropriate and is the work technically sound?

Yes

Are the conclusions drawn adequately supported by the results?

Partly

Are sufficient details of methods and analysis provided to allow replication by others?

Yes

Reviewer Expertise:

Cybersecurity, Information Security Management, Blockchain, Technology Adoption and E-Service

I confirm that I have read this submission and believe that I have an appropriate level of expertise to confirm that it is of an acceptable scientific standard, however I have significant reservations, as outlined above.

# Response

**Published:** 2022-09-13

**Authors:** Nachaat Mohamed

**Affiliations:** Rabdan Academy, United Arab Emirates

Dear respected reviewer Dr. Hamed,

 Thanks a lot for your valuable comments.

 Kindly find the amendments based on your comments and recommendations for the version (2) are below:

 - MITRE, ATT&CK, CALDERA, Red Team has been added to the Keyword section.

 - The punctuation has been added as you recommended in the sentence "In this study (In this study,) we used "54ndc47" agent through GoLang;

 - Grammatical error in "Microsoft have (has)"  It has been changed.

 - The limitation section has been added to the paper.

 - A brief introduction about AI has been added to the introduction section.

 - The motivation behind using MITRE CALDERA has been added at the end of the introduction section.

 - The new directions of the paper and future work has been added at the end of the conclusion section.

 - The difference between this paper and the rest papers in the related work section has been added at the end of the related work section.

 - References [49]-[50] has been added.

 Kind regards,

 Dr. Nachaat

# Peer review: Reviewer response for version 2

**DOI:** 10.5256/f1000research.134330.r139002

**Published:** 2022-06-29

**Authors:** Abdullah Alabdulatif

**Affiliations:** 1 Department of Computer, College of Sciences and Arts in Al-Rass, Qassim University, Al-Rass, Saudi Arabia

I have no further comments to make.

Is the work clearly and accurately presented and does it cite the current literature?

Partly

If applicable, is the statistical analysis and its interpretation appropriate?

Not applicable

Are all the source data underlying the results available to ensure full reproducibility?

Yes

Is the study design appropriate and is the work technically sound?

Yes

Are the conclusions drawn adequately supported by the results?

Yes

Are sufficient details of methods and analysis provided to allow replication by others?

Yes

Reviewer Expertise:

information security

I confirm that I have read this submission and believe that I have an appropriate level of expertise to confirm that it is of an acceptable scientific standard.

# Peer review: Reviewer response for version 1

**DOI:** 10.5256/f1000research.120614.r134931

**Published:** 2022-04-27

**Authors:** Abdullah Alabdulatif

**Affiliations:** 1 Department of Computer, College of Sciences and Arts in Al-Rass, Qassim University, Al-Rass, Saudi Arabia

The author has done very good work and explained the problem, which is an Advanced Persistent Threat (APT) against Microsoft Windows Security with the last versions of Windows 10 &11. In this part of the paper, the author has studied and analyzed the problem clearly. However, there are some references that are a bit old. They should be updated with some references in this part.

 After that, the author explains the methodology for attaching APT and supports the explanation with great steps and pictures that make this part easy to follow and understand for the reader. Before the end, the author presented the results and discussed them, along with some pictures to prove these results and identify flaws in the APT. I recommend the author discuss the results in depth, as well as make the discussion more clear. Finally, the conclusion part should be expanded by adding more about the future work.

Is the work clearly and accurately presented and does it cite the current literature?

Partly

If applicable, is the statistical analysis and its interpretation appropriate?

Not applicable

Are all the source data underlying the results available to ensure full reproducibility?

Yes

Is the study design appropriate and is the work technically sound?

Yes

Are the conclusions drawn adequately supported by the results?

Yes

Are sufficient details of methods and analysis provided to allow replication by others?

Yes

Reviewer Expertise:

information security

I confirm that I have read this submission and believe that I have an appropriate level of expertise to confirm that it is of an acceptable scientific standard, however I have significant reservations, as outlined above.

# Response

**Published:** 2022-04-30

**Authors:** Nachaat Mohamed

**Affiliations:** Rabdan Academy, United Arab Emirates

I would like to thank the reviewer for the valuable comments and I assure that first, I will change the old reference for the year 2010 with a new one. Secondly, I will add the future work to the conclusion section as recommended.

 Thank you
